# Development of [^124/125^I]IAZA as a New Proteinopathy Imaging Agent for Alzheimer’s Disease

**DOI:** 10.3390/molecules28020865

**Published:** 2023-01-15

**Authors:** Thrisha T. Reddy, Michael H. Iguban, Lusine L. Melkonyan, Jasmine Shergill, Christopher Liang, Jogeshwar Mukherjee

**Affiliations:** Preclinical Imaging, Department of Radiological Sciences, University of California-Irvine, Irvine, CA 92697, USA

**Keywords:** β-amyloid plaques, Alzheimer’s disease, imaging, transgenic 5xFAD mice, postmortem human AD brain, iodine-124, iodine-125, IAZA

## Abstract

Radioiodinated imaging agents for Aβ amyloid plaque imaging in Alzheimer’s disease (AD) patients have not been actively pursued. Our previous studies employed the “diaza” derivatives [^11^C]TAZA and [^18^F]flotaza in order to develop successful positron emission tomography (PET) imaging agents for Aβ plaques. There is a need for radioiodinated imaging agents for Aβ plaques for single photon emission computed tomography (SPECT) and PET imaging. We report our findings on the preparation of [^124/125^I]IAZA, a “diaza” analog of [^11^C]TAZA and [^18^F]flotaza, and the evaluation of binding to Aβ plaques in the postmortem human AD brain. The binding affinity of IAZA for Aβ plaques was Ki = 10.9 nM with weak binding affinity for neurofibrillary tangles (Ki = 3.71 μM). Both [^125^I]IAZA and [^124^I]IAZA were produced in >25% radiochemical yield and >90% radiochemical purity. In vitro binding of [^125^I]IAZA and [^124^I]IAZA in postmortem human AD brains was higher in gray matter containing Aβ plaques compared to white matter (ratio of gray to white matter was >7). Anti-Aβ immunostaining strongly correlated with [^124^/^125^I]IAZA in postmortem AD human brains. The binding of [^124^/^125^I]IAZA in postmortem human AD brains was displaced by the known Aβ plaque imaging agents. Thus, radiolabeled [^124/123^I]IAZA may potentially be a useful PET or SPECT radioligand for Aβ plaques in brain imaging studies.

## 1. Introduction

The prevalence of dementia is vastly increasing due to a rising older population. The primary form of dementia in the aging population is Alzheimer’s disease (AD), which accounts for 80% of all dementia. Alzheimer’s disease is a proteinopathy characterized by the accumulation of amyloid β (Aβ) plaques and the Tau protein, causing neurofibrillary tangles (NFT) in the brain [1,2]. Molecular biomarkers are now becoming indispensable for clinical evaluation of AD [3,4,5]. Imaging studies for Aβ plaques and Tau in AD have been robustly pursued using various carbon-11 and fluorine-18 labeled positron emission tomography (PET) agents. Limited effort has been made on radioiodine Aβ plaques and Tau imaging agents for single photon emission computerized tomography (SPECT) studies.

**Figure 1:**
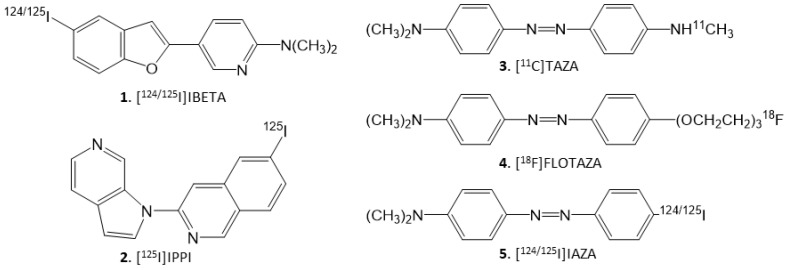
Chemical structures of Aβ-plaque- and Tau-binding radioligands: **1.** [^124/125^I]IBETA; **2**. [^125^I]IPPI; **3.** [^11^C]TAZA; **4.** [^18^F]Flotaza; **5.** [^124/125^I]IAZA.

**Figure 2:**
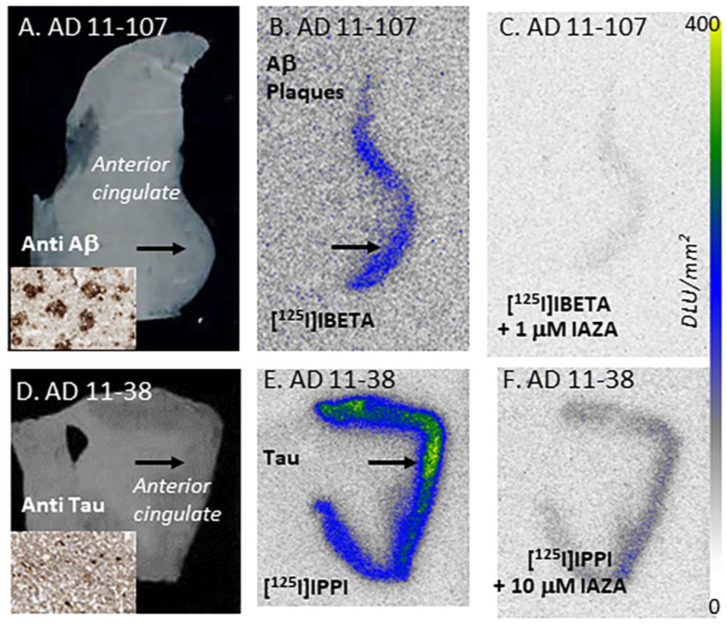
In vitro competition of IAZA with [^125^I]IBETA and [^125^I]IPPI in postmortem human AD brain anterior cingulate (AC) and corpus callosum. (**A**) Brain slice of postmortem human AD 10 µm thick (inset shows scan anti-Aβ IHC labeled AC). (**B**) Adjacent slice of AC of same AD subject showing total binding of [^125^I]IBETA in Aβ-rich regions (arrows). (**C**) Same AD subject in the presence of 1 μM IAZA showing displacement of [^125^I]IBETA from AC. (**D**) Brain slice 10 µm thick of postmortem human AD subject (inset shows scan anti-Tau IHC labeled AC). (**E**) Adjacent slice of AC of same AD subject showing total binding of [^125^I]IPPI binding in Tau-rich regions (arrows); (**F**). Same AD subject in the presence of 10 μM IAZA showing partial displacement of [^125^I]IPPI from AC.

The underlying cause of proteinopathy in AD remains unknown, and imaging proteinopathies in the brain may be helpful for AD risk prediction, diagnosis of cerebral amyloidosis and evaluating the efficacy of anti-amyloid treatments. PET studies of Aβ plaque accumulation in AD have shown clinical utility [6,7,8,9]. Successful clinical research trials utilizing several PET radiotracers are now being pursued for both Aβ plaques and Tau NFT [10]. Studies using SPECT for Aβ plaque imaging have not advanced as much as PET studies [11]. Radioiodinated derivatives using iodine-125 and iodine-123 for imaging Aβ plaques have been pursued by several investigators [12,13]. Promising preliminary human SPECT imaging studies using [^123^I]ABC577 were reported and compared with known PET imaging agents [14]. We have recently reported promising brain biodistribution studies in 5xFAD transgenic mouse models as well as in postmortem human AD brain studies using [^124^I]IBETA (Figure 1, **1**) [15]. Iodinated phenyldiazenyl benzothiazole derivatives have been reported for imaging NFT in AD brains [16]. Our recent work on the selective azaindole [^125^I]IPPI (Figure 1, **2**) for imaging human Tau NFT shows promise as a potential agent for SPECT imaging when labeled with iodine-123. [17].

**Figure 3:**
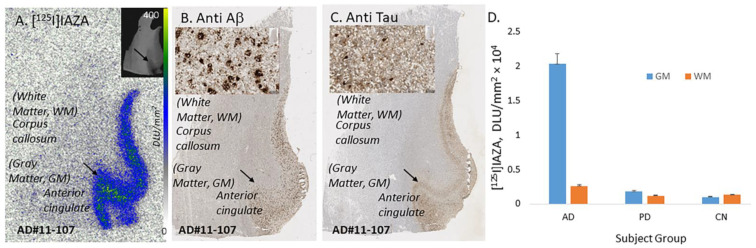
In vitro binding of [^125^I]IAZA in human postmortem AD, PD and CN brains consisting of anterior cingulate. (**A**) Postmortem human AD 10 µm thick brain slice shows [^125^I]IAZA binding mainly in the gray matter (GM) regions of anterior cingulate (AC) and none in the white matter (WM) regions of corpus callosum (CC). (**B**) Adjacent slice of the same subject immunostained with anti-Aβ showing abundance of Aβ plaques in the GM regions (inset shows magnified view of Aβ plaques). (**C**) Adjacent slice of the same subject immunostained with anti-Tau showing abundance of Tau in the GM regions (inset shows magnified view of Tau). (**D**) Plot shows high binding of [^125^I]IAZA in gray matter (GM) and white matter (WM) regions of AD subjects, while PD and CN subjects had little binding.

**Figure 4:**
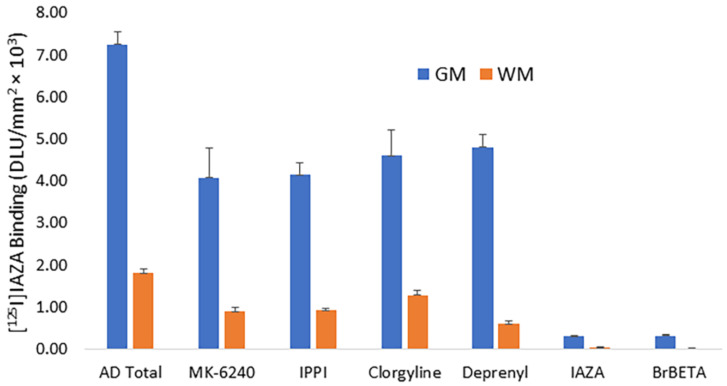
In vitro competition of [^125^I]IAZA with drugs in postmortem human AD brains containing temporal cortex and anterior cingulate showing binding in the gray matter (GM) and white matter (WM). All drugs used were at 10 μM concentration. Tau-binding drugs MK-6240 and IPPI reduced the binding to approximately 55%. Both MAO-A drug clorgyline and MAO-B drug deprenyl reduced the binding to approximately 65%. Drugs binding to Aβ plaques, IAZA and BrBETA, were able to displace most of the [^125^I]IAZA binding (>95%).

Our previous work on [^11^C]TAZA (Figure 1, **3**), which contains heteroatoms in the “diazo” functionality, resulted in higher affinity to Aβ plaques and lower white matter binding in postmortem human AD brain slices [18]. Similarly, we developed a fluorine-18 analog of [^11^C]TAZA namely [^18^F]Flotaza (Figure 1, **4**) and evaluated it for Aβ plaque imaging [19]. [^18^F]Flotaza yielded significantly higher binding in the gray matter regions, where Aβ plaques were located and which were confirmed by anti-Aβ immunostains. The additional “diazo” heteroatoms render [^11^C]TAZA and [^18^F]Flotaza less lipophilic, resulting in lower white matter binding, thus providing high gray matter to white matter ratios in the in vitro postmortem human brain slices [18,19]. 

**Figure 5:**
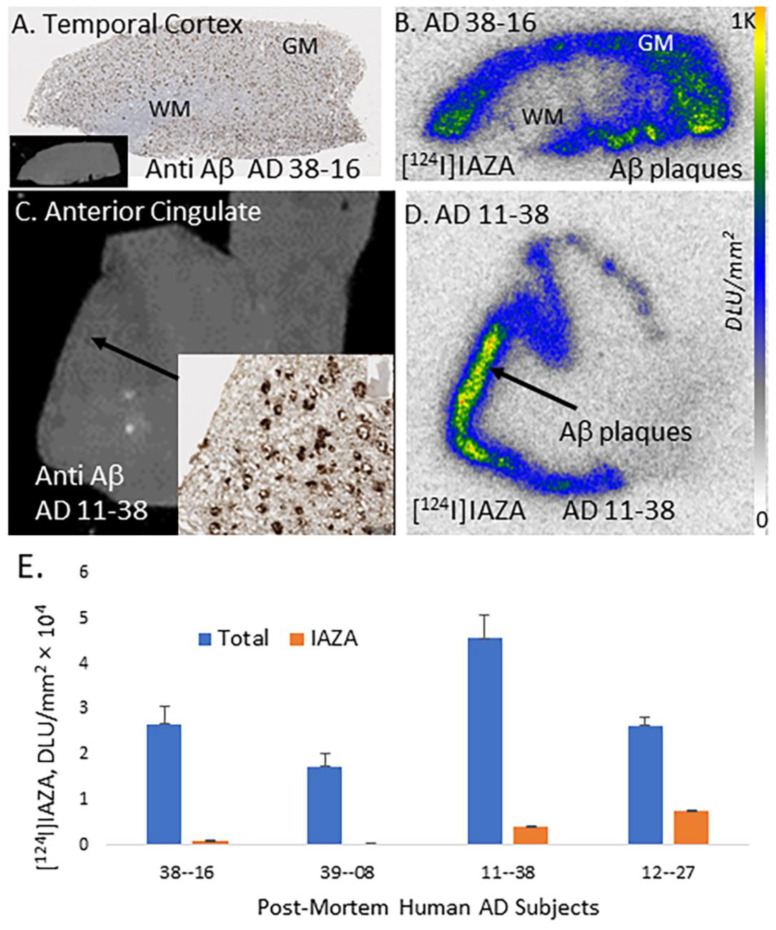
In vitro binding of [^124^I]IAZA in postmortem human AD brain. (**A**) Anti-Aβ IHC postmortem human AD 10 µm thick brain slice of temporal cortex (inset shows scan of brain slice). (**B**) Adjacent slice of temporal cortex of same AD subject showing [^124^I]IAZA binding in Aβ rich regions. (**C**) Human AD anterior cingulate (AC) and corpus callosum (CC) in 10 µm brain slice, inset shows anti-Aβ IHC in AC, where most Aβ plaques are located. (**D**) Adjacent slice of same AD subject showing [^124^I]IAZA binding in Aβ-rich regions in AC and none in CC. (**E**) A plot shows high [^124^I]IAZA binding in cortical regions of AD subjects, which was displaced by 10 μM of unlabeled IAZA.

Because of this unique property of the “diazo” feature, our goal in this study was to use the “diazo” functionality in order to prepare a radioiodinated imaging agent for AD. We identified [^124/125^I] 4′-iodo-4-dimethylaminoazobenzene (acronym: [^124/125^I]IAZA) (Figure 1**, 5**) as a potential lead structure which contains the 4-*N*,*N*-dimethylamino feature similar to [^11^C]TAZA and [^18^F]Flotaza, along with the “diazo” functionality. Both [^11^C]TAZA and [^18^F]Flotaza bind to Aβ plaques, thus the anticipation was that [^124/125^I]IAZA would bind Aβ plaques., A previous report on a different class of “diazo” derivatives indicated some targeting to Tau [16]. Thus, our goal here was to carry out the following: (a) radiosynthesis of [^124/125^I]IAZA; (b) assessment of binding affinity of IAZA to Aβ plaques and Tau; (c) evaluation of the binding of [^125^I]IAZA in postmortem human AD brain slices in vitro; (d) evaluation of drug effects on the binding of [^125^I]IAZA in postmortem human AD brain slices in vitro; (e) carrying out of a preliminary evaluation of [^124^I]IAZA in postmortem human AD brain slices in vitro. Iodine-124 (longer half-life of 4.2 days) labeled PET imaging agents may be suitable for extended PET imaging [20]. Additionally, since PET has a higher resolution compared to SPECT, it may be useful for the identification of small brain regions in transgenic AD mice brains with Aβ plaque accumulation [15].

## 2. Results

### 2.1. Synthesis 

The tributyltin precursor **7** was prepared in good yield and was found to be stable for radioiodination. Similar to our previously published procedures [15], hydrogen peroxide (H_2_O_2_) was used as an oxidant in the radiolabeling with [^125^I]NaI and [^124^I]NaI. At room temperature, the radiolabeling reactions with [^125^I]NaI and [^124^I]NaI proceeded for 30 min before being terminated. The purification and isolation of [^125^I]IAZA and [^124^I]IAZA using preparative TLC was efficient, with little loss on the silica. The isolated products showed a purity of >95%, as indicated by radio TLC with a radiochemical yield of approximately 25–30%. Both [^125^I]IAZA and [^124^I]IAZA were found to be stable in ethanolic solutions and used for in vitro studies.

### 2.2. Binding Affinity Studies

Increasing concentrations of IAZA (1 nM to 10 μM) progressively displaced [^125^I]IBETA, with 1 μM IAZA displacing >90% of [^125^I]IBETA bound to the Aβ plaques, as shown in Figure 2C. An IC_50_ = 15.8 nM was measured for IAZA binding to Aβ plaques. Using the affinity of [^125^I]IBETA (2.36 nM [15]), the binding affinity Ki of IAZA for Aβ plaques was calculated to be 10.9 nM. In the case of Tau, increasing concentrations of IAZA (1 nM to 10 μM) had a lower effect on the binding of [^125^I]IPPI, with 10 μM IAZA displacing about 50% of [^125^I]IPPI bound to Tau, as shown in Figure 2F. An IC_50_ = 8.65 μM was measured for IAZA binding to Tau. Using the affinity of [^125^I]IPPI (0.75 nM [17]), the binding affinity Ki of IAZA for Tau was calculated to be 3.71 μM.

### 2.3. [^125^I]IAZA in Postmortem Human AD Brain

Postmortem human AD subjects (n = 6), PD subjects (n = 4) and CN subjects’ (n = 4) brain sections containing anterior cingulate (AC) were used to evaluate the total binding of [^125^I]IAZA (Figure 3). Binding of [^125^I]IAZA was higher in the gray matter (GM) consisting of AC, while little binding was observed in the white matter (WM) comprising corpus callosum (CC) (Figure 3A). The presence of Aβ plaques in the AC of all AD subjects was confirmed by anti-Aβ immunostaining (Figure 3B). Additionally, [^125^I]IAZA binding in the AC strongly correlated with anti-Aβ immunostains for Aβ plaques in the same subjects, hence confirming the specific affinity of [^125^I]IAZA to Aβ plaques in the AD brain (Figure 3A,B). It should be noted that the AC regions of the AD brain slices were also stained for Tau, as shown in Figure 3C, and overlapped with the anti-Aβ immunostain. The average ratio between AC to CC for AD subjects was 7.9 (Figure 3D), while for PD subjects AC/CC = 1.52 and for CN subjects AC/CC ≤ 1.

Drug effects on [^125^I]IAZA were carried out on brain slices of temporal cortex and AC from postmortem human AD subjects’ brains (Figure 4). In the absence of any drugs, binding of [^125^I]IAZA was higher in the gray matter, while binding in the white matter was lower (Figure 4, AD total average of both AC and temporal cortex). Upon the addition of Aβ-plaque-binding drugs (10 μM each), IAZA and BrBETA [15], more than 95% of [^125^I]IAZA binding to Aβ plaques was displaced, suggesting a high affinity for Aβ binding sites (Figure 4, IAZA and BrBETA). The Tau agents MK-6240 and IPPI [17] at 10 μM concentrations reduced the binding to approximately 55%. Similarly, MAO inhibitors clorgyline for MAO-A and deprenyl for MAO-B at 10 μM concentrations reduced the binding of [^125^I]IAZA to approximately 65% of the total binding.

### 2.4. [^124^I]IAZA in Postmortem Human AD Brain

Postmortem human AD (n = 4) brain sections of AC and temporal cortex were used to evaluate the binding of [^124^I]IAZA to Aβ plaques. All brain sections contained GM and WM regions. Shown in Figure 5A is the AD temporal cortex brain slice immunostained with anti-Aβ, which confirmed the presence of Aβ plaques in the GM while WM had none. Binding of [^124^I]IAZA in the Aβ-plaque-rich GM of the temporal cortex is seen in Figure 5B. This binding of [^124^I]IAZA was displaced by 10 μM IAZA, resulting in a total/nonspecific binding ratio of >20. Similarly, binding of [^124^I]IAZA in the GM regions of AC of AD subject 11-38 (Figure 5C). This subject had extensive Aβ plaques which was previously confirmed with [^18^F]flotaza [19]. Extensive [^124^I]IAZA was seen in the AC of AD 11-38 (Figure 5D) which strongly correlated with anti-Aβ immunostains for Aβ plaques (Figure 5C,D). The total GM binding to nonspecific WM binding ratio was >11.

The higher gamma energy photons of iodine-124 provided good autoradiographic imaging of [^124^I]IAZA labeled brain slices after an overnight exposure on phosphor films. In contrast, the weaker gamma energy of iodine-125 labeled [^125^I]IAZA took longer exposure times (typically a week) to provide sufficient quality autoradiographic images for quantitative analyses.

## 3. Discussion

In an effort to obtain high-affinity radioiodinated Aβ plaque imaging agents, we pursued different classes of compounds. Previously we reported on the development of [^124^I]IBETA, which exhibited good in vitro binding in postmortem human AD brain slices and 5xFAD transgenic mouse brain slices [15]. In addition, preliminary PET/CT imaging studies in 5xFAD mice using [^124^I]IBETA indicated promise, since it localized in brain regions rich in Aβ plaques [15]. Thus, more detailed in vivo PET/CT imaging studies using [^124^I]IBETA in 5xFAD mice are planned.

Because of the unique properties of the “diaza” functionality in giving high target-to-nontarget ratios as exhibited by [^18^F]flotaza [19], our interest was to examine a “diaza” radioiodinated derivative. We were circumspect about the target molecule IAZA, because it lacked a heteroatom at the para-position like flotaza or TAZA (Figure 1), and it was not known how that might have an adverse effect on its binding affinity for Aβ plaques. Our results show that it indeed had a somewhat weaker affinity (Ki = 10.9 nM) compared to flotaza (Ki = 1.68 nM, [19]) and IBETA (Ki = 2.36 nM, [15]), but is still sufficient to carry out in vitro binding studies. A second uncertain factor was the affinity of IAZA towards Tau, because previously a “diaza” derivative was shown to have significant affinity for Tau [16]. Our measures of the affinity of IAZA for Tau show it is weak, with micromolar affinities (Ki = 3.71 μM). Thus, in the radiotracer studies presented here, where nanomolar concentrations of [^124/125^I]IAZA were used, the predominant binding observed was to Aβ plaques. Because IAZA has a somewhat weaker affinity for Aβ plaques compared to IBETA, TAZA and flotaza, in vivo imaging efficacy of [^124^I]IAZA remains to be determined.

Since the radiosynthesis involved acidic oxidative conditions for radioiodination, there was a concern about the stability of the “diaza” linkage. However, the use of the tributyltin derivative proved effective in providing reasonable yields of both [^125^I]IAZA and [^124^I]IAZA with high radiochemical purity. Ethanolic solutions of both radiotracers were stable when stored at −20 °C.

Binding of [^125^I]IAZA to Aβ plaques in AD brain slices correlated with anti-Aβ immunostains, suggesting the high affinity of this radioligand to Aβ plaques in the AD brain. White matter showed nonspecific binding and was variable in different brain samples. The washing protocol which uses alcohol may have to be further optimized in order to obtain a more consistent nonspecific binding. As expected, PD and CN brains which did not contain Aβ plaques did not reveal any significant levels of [^125^I]IAZA GM binding. It should be noted that [^18^F]flotaza had minimal WM binding in the AD brain slices in vitro, which may be due to the polyethylene glycol functionality in the structure [19].

Drug effects were assessed in order to further verify the binding of [^125^I]IAZA to Aβ plaques in the postmortem human AD brain slices. As expected, Aβ-plaque-binding drugs IAZA and BrBETA [15] blocked more than 95% of [^125^I]IAZA binding to Aβ plaques. At high concentrations (10 μM) of the Tau-binding drugs, MK-6240 and IPPI [17], and MAO inhibitors, clorgyline and deprenyl, the binding of [^125^I]IAZA was reduced to approximately 55–65% of the total binding. These findings suggest that [^125^I]IAZA is predominantly binding to the Aβ plaques.

In anticipation of undertaking future in vivo studies for the evaluation of Aβ drugs [21] and testing using other proteinopathy animal models [22,23,24], [^124^I]IAZA was successfully prepared. Excellent binding of [^124^I]IAZA was observed in the AD brain temporal cortex as well as the AC. The regional localization of both [^125^I]IAZA and [^124^I]IAZA in the same subjects was similar, although the higher energy of the iodine-124 photons compared to the iodine-125 resulted in more intense [^124^I]IAZA autoradiographic images. Because [^124^I]IAZA will be used as a radiotracer, less than one microgram of mass will be administered for imaging studies. Therefore, no pharmacological effect may be expected. However, because of the presence of radioactive iodine in the molecule, in vivo studies will have to be performed to confirm brain uptake, metabolism and clearance from different organs. In addition, the amount of thyroid uptake will have to be confirmed if [^124^I]IAZA is broken down and releases free radioactive iodine.

In medical centers that may not have ready access to PET scanners, SPECT imaging, being less expensive, can provide an alternative to Aβ plaque imaging. Iodine-123 labeled SPECT imaging (DaTscan) is currently used clinically in Parkinson’s disease [25]. With the increase in the number of AD patients, and a limited number of PET centers, the availability of a SPECT imaging agent can have a significant impact. The availability of SPECT imaging agents will allow Nuclear Medicine clinics, who may not have PET capability, to purchase the iodine-123 labeled radiopharmaceutical for imaging studies. In addition, the availability of iodine-124 labeled radiopharmaceuticals will allow flexibility of transportation to remote PET sites. Iodine-124, because of its longer half-life, will allow for extended imaging times, enabling greater clearance of nonspecific binding and thus providing potentially better image contrast, which may be useful when small levels of Aβ plaques are present.

## 4. Materials and Methods

### 4.1. General Methods

All chemicals and solvents were purchased from Aldrich Chemicals, Fisher Scientific, and 1Click Chemistry (4′-iodo-4-dimethylaminoazobenzene). Deionized water was acquired from Millipore Milli-Q Water Purification System. Iodine-125 sodium iodide, carrier-free (specific activity = 643 MBq/μg) in 0.01N NaOH, was purchased from American Radiolabeled Chemicals, Inc., St. Louis, MO, USA. Iodine-124 sodium iodide, carrier-free (specific activity ≥ 1000 GBq/μmole) in 0.01N NaOH, was purchased from 3DImaging, LLC., Little Rock, AK, USA. Iodine-124 and iodine-125 radioactivities were counted in a Capintec CRC-15R dose calibrator, while low-level counting was carried out in a Capintec Caprac-R well-counter. Gilson high-performance liquid chromatography (HPLC) was used for the semi-preparative reverse-phase column chromatography with UV detector set at dual wavelengths of 254 nm and 280 nm, as well as a radioactivity detector. A semi-preparative HPLC column 10 × 250 mm Econosil C18 reverse-phase was used. Analytical thin-layer chromatography (TLC) was used to monitor reactions (Baker-flex, Phillipsburg, NJ, USA). RadioTLC was scanned on an AR-2000 imaging scanner (Eckart & Ziegler, Berlin, Germany). Electrospray mass spectra were obtained from a Model 7250 mass spectrometer (Micromass LCT). Proton NMR spectra were recorded on a Bruker OM EGA 500-MHz spectrometer. Brain slices were prepared in 10 µm thick slices using the Leica 1850 cryotome. In vitro labeled brain sections were exposed to phosphor films (Perkin Elmer Multisensitive, Medium MS) and read using the Cyclone Phosphor Imaging System (Packard Instruments). Analysis of in vitro autoradiographs was carried out by using the Optiquant acquisition and analysis software (Packard Instruments). Immunostains were analyzed using QuPath.

### 4.2. Human Tissue

Human postmortem brain tissue samples were obtained from Banner Sun Health Research Institute (BHRI), Sun City, AZ, and UCI MIND brain tissue repository for in vitro experiments. Brain tissue samples from anterior cingulate of AD, Parkinson’s disease (PD) brain and cognitively normal (CN) brain tissue samples were obtained from BHRI. Brain tissues comprising of temporal cortex from AD subjects were obtained from UCI MIND repository. Chunks of frozen tissue were sectioned on a Leica 1850 cryotome cooled to −20 °C, collected on Fisher slides and subsequently stored at −80 °C. Iodine-124 and iodine-125 autoradiographic studies were carried out by exposing tissue samples on storage phosphor screens. Adjacent slices were used for immunostaining with anti-Aβ. All experiments were carried out in accordance with the University of California, Irvine, and were consistent with federal guidelines. All postmortem human brain studies were approved by the Institutional Biosafety Committee of University of California, Irvine. 

### 4.3. Synthesis 

**Figure 6:**
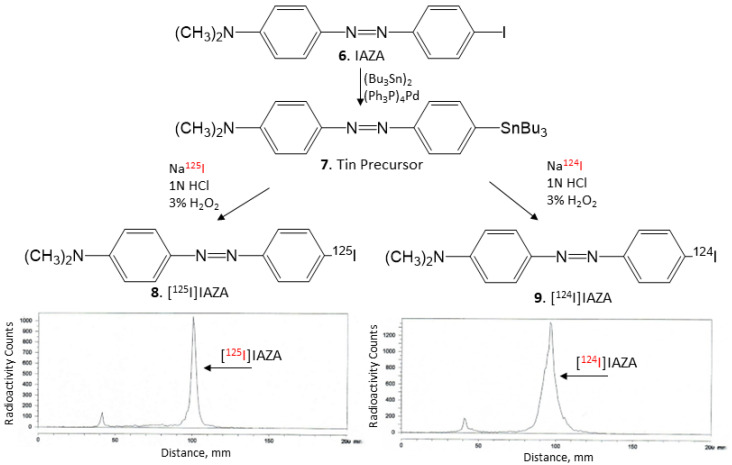
[^125^I]NaI and [^124^I]NaI were used to prepare [^125^I]IAZA **(8)** and [^124^I]IAZA **(9)** by electrophilic substitution of the tributyltin derivative. TLC of [^125^I]IAZA shows the purity of >90%, and TLC of [^124^I]IAZA shows the purity of >90%.

Nonradioactive IAZA (Figure 6, **6**) (35 mg, 0.1 mmol) was treated with bis(tributyltin) (58 mg, 0.1 mmol) in the presence of (Ph_3_P)_4_Pd (5.5 mg, 0.005 mmol) in a dioxane/triethylamine solvent mixture (0.8 mL, 3:1). The mixture was refluxed at 90 °C overnight. The reaction mixture was purified using preparative silica gel TLC in hexane/ethyl acetate (1:1). The purified tin precursor **7** was obtained in 20% yield. ^1^H NMR (CDCl_3_, 500 MHz) δ ppm: 7.90–7.82 (m, 4H), 7.02–7.0 (d, 2H), 6.80–6.78 (d, 2H), 3.10 (s, 6H, N(CH_3_)_2_), 1.60–1.38 (m, 12H), 1.26 (m, 6H), 0.92 (t, 9H). Mass Spectra (ESI): m/z 516 ([M + H]^+^, 100%).

### 4.4. Radiosynthesis 

Using our reported radioiodination methods for IBETA [15], sodium iodide, [^125^I]NaI (ARC Inc.) and [^124^I]NaI (3D Imaging LLC) were used to prepare [^125^I]IAZA and [^124^I]IAZA by electrophilic substitution of the tributyltin derivative **7**. For synthesis of [^125^I]IAZA, tributyltin derivative (0.1 mg/0.1 mL of ethanol) and 5 MBq [^125^I]NaI were used. To the reaction vial containing the tin precursor and [^125^I]NaI, 0.1 mL H_2_O_2_ (3%) was added followed by 0.1 mL of 1N HCl in a sealed vial. The reaction was allowed to proceed at room temperature for 30 min before it was terminated by the addition of sodium bisulfite 0.1 M. The purification and isolation of [^125^I]IAZA were conducted on preparative TLC and the product was extracted with ethanol. RadioTLC confirmed radiochemical purity of >90% [^125^I]IAZA (Figure 6, **8**). Molar activity was estimated to be approximately 90 TBq/mmole under the no-carrier-added conditions. Purified [^125^I]IAZA was stored at −20 °C as an ethanolic solution and was found to be stable for several months, ascertained by radioTLC.

For [^124^I]IAZA, similar methods as described above were successfully used. To a mixture of 0.1 mL of tributyltin derivative (1 mg/0.1 mL of ethanol), 25 MBq [^124^I]NaI, 0.1 mL of 1N HCl and 0.1 mL H_2_O_2_ (3%) were added in a sealed vial. The reaction was allowed to proceed at room temperature for 30 min before it was terminated by the addition of sodium bisulfite. The reaction mixture was extracted using dichloromethane and [^124^I]IAZA was purified on preparative TLC and extracted with ethanol. RadioTLC confirmed radiochemical purity of >90% [^124^I]IAZA (Figure 6, **9**). Molar activity was estimated to be >500 TBq/mmole under the no-carrier-added conditions. Purified [^124^I]IAZA in an ethanolic solution was used on the same day for in vitro studies.

### 4.5. In Vitro Postmortem Human Brain Autoradiography

Brain slices (10 μm thickness) on Fisher slides were taken out of a −80 °C freezer on the day of the experiment and were allowed to thaw to room temperature. The slides contained one to four brain sections each and were placed in separate glass chambers (six slides per chamber). The slides were preincubated in PBS buffer for 15 min at ambient temperature. The preincubation buffer was discarded. The brain slices were then treated with [^124^I]IAZA or [^125^I]IAZA in 40% ethanol PBS buffer pH 7.4 (60 mL, 5 kBq/mL). The chambers were incubated at 25 °C for 1.25 h. Nonspecific binding was measured in separate chambers using IAZA (10 µM). The slices were then washed with cold PBS buffer, 90% ethanolic PBS buffer twice, PBS buffer and cold water. The brain sections were air-dried and exposed on a phosphor film (Multisensitive Medium MS, PerkinElmer, Waltham, MA). The apposed phosphor screens were read and analyzed by OptiQuant acquisition and the Cyclone Storage Phosphor System (Packard Instruments Co., Boston, MA). Regions-of-interest (ROI) were drawn on the slices containing gray matter (GM) and white matter (WM) areas and the extent of binding of [^124^I]IAZA and [^125^I]IAZA was measured in DLU/mm^2^. 

Drug effects on [^125^I]IAZA included IAZA and BrBETA, which bind to Aβ plaques [15], and MK-6240 and IPPI, which are known to bind to human Tau [17]. Competition with monoamine oxidase inhibitors clorgyline (MAO-A) and deprenyl (MAO-B) was also carried out, since MAO has been a potential off-target site for some proteinopathy imaging agents, as discussed in our previous work with [^125^I]IPPI [17]. Each of the drugs were used at a concentration of 10 μM.

### 4.6. Binding Affinity Studies

Autoradiographic methods were used to measure binding affinities, similar to our previously described work [26]. Competitive binding assays with different concentrations of IAZA (1 nM to 10 μM) were carried out using human brain slices under the conditions described above. For measurement of affinity to Aβ plaques, competition of IAZA was carried out with [^125^I]IBETA (1 nM) in various regions in human AD brain sections and 5xFAD mice. Nonspecific binding was measured using BrBETA [15]. Data were analyzed using the following procedure: (1) the non-specific binding of [^125^I]IBETA was subtracted for all samples; (2) the specific binding was normalized to 100% (no competitive ligand); and (3) the binding isotherms were fit to the Hill equation (KELL BioSoft software (v 6), Cambridge, U.K.) to provide inhibitor concentration (IC_50_) which was the inflection point of the isotherm where 50% of the [^125^I]IBETA binding was displaced. Affinity (Ki) was calculated using the Cheng–Prusoff equation.

For the Tau binding assay, [^125^I]IPPI (1 nM) was used with different concentrations of IAZA (1 nM to 10 μM) in human AD brain slices and nonspecific binding was measured using IPPI [17]. Data were analyzed using the following procedure: (1) the non-specific binding of [^125^I]IPPI was subtracted for all samples; (2) the specific binding was normalized to 100% (no competitive ligand); and (3) the binding isotherms were fit to the Hill equation (KELL BioSoft software (v 6), Cambridge, U.K.) to provide inhibitor concentration (IC_50_), which was the inflection point of the isotherm where 50% of the [^125^I]IPPI binding was displaced. Affinity (Ki) was calculated using the Cheng–Prusoff equation.

### 4.7. Immunohistochemistry

Immunostaining of all brain sections were carried out by University of California-Irvine, Pathology Services using Ventana BenchMark Ultra protocols. To determine the localization of Tau in the human AD brain sections, neighboring slices were immunostained with the DAKO polyclonal antibody, which binds to total Tau which detects all 6 six isoforms of Tau (dilution 1: 3000, A0024; Agilent, CA, USA). For Aβ plaques, neighboring slices were immunostained with anti-Aβ Biolegend 803015 (Biolegend, CA, USA), reactive to amino acid residue 1–16 of Aβ. Immunostained sections were scanned using the Ventana Roche instrumentation and the images were analyzed using QuPath software.

### 4.8. Image Analysis

Statistical differences between groups (AD, CN and PD) were determined using Microsoft Excel 16. Statistical power was determined with Student’s *t* test and *p* < 0.05 was considered to be significant.

## 5. Conclusions

In summary, [^124/125^I]IAZA is a new Aβ PET imaging agent which can be made in a one-step radiosynthesis and was found to be stable. It exhibited high binding to Aβ plaques in postmortem human AD brain tissues in vitro. Our future plans are to evaluate in vitro and in vivo binding of [^124^I]IAZA in 5xFAD transgenic AD mice in order to establish brain permeability, metabolic stability and selectivity of binding to Aβ plaques. These findings will be compared with our previous results of [^124^I]IBETA. If successful, SPECT imaging studies in 5xFAD mice using [^123^I]IAZA may be possible.

## Data Availability

The data that support the findings of this study are available from the corresponding author upon reasonable request.
